# A Plane-Dependent Model of 3D Grid Cells for Representing Both 2D and 3D Spaces Under Various Navigation Modes

**DOI:** 10.3389/fncom.2021.739515

**Published:** 2021-09-22

**Authors:** Ziyi Gong, Fangwen Yu

**Affiliations:** ^1^Center for Brain Inspired Computing Research, Tsinghua University, Beijing, China; ^2^Department of Neurobiology, School of Medicine, Duke University, Durham, NC, United States

**Keywords:** grid cell, space representation, path integration, navigation, two-dimensional space, three-dimensional space

## Abstract

Grid cells are crucial in path integration and representation of the external world. The spikes of grid cells spatially form clusters called grid fields, which encode important information about allocentric positions. To decode the information, studying the spatial structures of grid fields is a key task for both experimenters and theorists. Experiments reveal that grid fields form hexagonal lattice during planar navigation, and are anisotropic beyond planar navigation. During volumetric navigation, they lose global order but possess local order. How grid cells form different field structures behind these different navigation modes remains an open theoretical question. However, to date, few models connect to the latest discoveries and explain the formation of various grid field structures. To fill in this gap, we propose an interpretive plane-dependent model of three-dimensional (3D) grid cells for representing both two-dimensional (2D) and 3D space. The model first evaluates motion with respect to planes, such as the planes animals stand on and the tangent planes of the motion manifold. Projection of the motion onto the planes leads to anisotropy, and error in the perception of planes degrades grid field regularity. A training-free recurrent neural network (RNN) then maps the processed motion information to grid fields. We verify that our model can generate regular and anisotropic grid fields, as well as grid fields with merely local order; our model is also compatible with mode switching. Furthermore, simulations predict that the degradation of grid field regularity is inversely proportional to the interval between two consecutive perceptions of planes. In conclusion, our model is one of the few pioneers that address grid field structures in a general case. Compared to the other pioneer models, our theory argues that the anisotropy and loss of global order result from the uncertain perception of planes rather than insufficient training.

## 1. Introduction

Navigation is crucial for animals to survive in nature. Animals have to navigate for foraging, exploring environments, and mating. During navigation, animals need to be aware of their allocentric self-positions by integrating their self-motion and other somatosensory information, a process known as path integration (Darwin, 1873; Etienne and Jeffery, 2004; McNaughton et al., 2006). Grid cells in mammalian medial entorhinal cortex (mEC) are crucial for this process, representing space like a coordinate system (McNaughton et al., 2006; Fiete et al., 2008; Horner et al., 2016; Gil et al., 2018; Ridler et al., 2020). The spatial firing fields of grid cells, called grid fields, form hexagonal lattice during horizontal planar navigation (Hafting et al., 2005; Fyhn et al., 2008; Doeller et al., 2010; Yartsev et al., 2011; Killian et al., 2012; Jacobs et al., 2013). It is then natural to inquire about the grid fields beyond planar navigation, as animals live in a three-dimensional (3D) world and carry multiple modes of navigation–planar, multilayered, and volumetric (Finkelstein et al., 2016). Because hexagonal lattice is the most efficient packing in two-dimensional (2D) space, a reasonable prediction is one of the maximally efficient 3D structures: face-centered cubic (FCC) or hexagonal-close-packed (HCP) (Gauss, 1840; Mathis et al., 2015).

However, recordings of grid cells on sloped, multilayered and volumetric environments (Hayman et al., 2011, 2015; Casali et al., 2019; Kim and Maguire, 2019; Ginosar et al., 2021; Grieves et al., 2021) challenge the theoretical prediction of isotropic 3D grid fields. Hayman et al. (2011) uncovered that when navigating on a pegboard and a helix, rats manifest vertically elongated grid fields; this anisotropy suggests columnar fields (COL) in 3D. In the subsequent research, rat grid fields on slopes (Hayman et al., 2015) and a vertical wall (Casali et al., 2019) could fit less-organized hexagonal lattice, instead of oblique slices of FCC or HCP. In other words, grid cell computation is projected onto the 2D arenas. During volumetric navigation, grid fields at least preserve local order. Two latest studies on rats navigating in a cubic lattice and bats flying in a rectangular room report that the volumetric grid fields do not fit into HCP, FCC, or COL, but are more regular than a random organization of fields (RND) (Ginosar et al., 2021; Grieves et al., 2021). On the contrary, another study implies that FCC could explain the fMRI activities of the entorhinal cortex of human subjects navigating in a 3D virtual reality paradigm (Kim and Maguire, 2019). Together, the available reports point to the presence of multimodality in grid codes, corresponding to the various and complicated forms of navigation in reality (Finkelstein et al., 2016).

A theoretical study is crucial to summarize the findings and explicate how grid cells represent the space during different navigation modes. To date, there lacks an interpretive model that encompasses all the latest experimental observations, despite the theoretical efforts predicting the neural basis of hexagonal lattice during planar navigation (Kropff and Treves, 2008; Burak and Fiete, 2009; D'Albis and Kempter, 2017; Banino et al., 2018; Cueva and Wei, 2018; Soman et al., 2018; Gao et al., 2019, 2021; Zeng et al., 2019). Previously, three volumetric grid cell models were raised. One of them does not require training; it contains ring attractors that generate stripes, and the combination of them gives rise to spherical fields (Horiuchi and Moss, 2015). But this approach does not account for the loss of global order. On the contrary, Stella and Treves (2015) and Soman et al. (2018) proposed two plasticity-based models, where training is necessary. In both of the works, the training is massive, while it is undetermined whether regular grid fields need the training to a comparable degree. More importantly, they attribute the loss of grid field regularity to insufficient training. Yet, insufficient training may be overcome by the dynamic learning capacity of the nervous system (refer to Discussion). As such, we alternatively focus on perception and cognition, another possible source of regularity degradation.

To explain the observed grid field structures, we propose an overarching theory on the grid cell computation in 3D, and implement the theory with a training-free recurrent neural network (RNN) extended from (Gao et al., 2021). The model is capable of representing both 2D and 3D spaces during various navigation modes. Specifically, it contains a representational model of motion and an RNN generating grid cell activities. The representational model interprets navigation with respect to real or virtual planes. For instance, the planes can be the supportive planes that navigators stand on or the tangents planes of the motion manifold. At each small time window, the displacement is decomposed in to two components: one on the corresponding plane, and the other is perpendicular to the plane. Then, the RNN updates itself with the weights dependent on the two components. We prove that the recurrent update is rotational and gives rise to spatial periodicity. With a proper biologically meaningful choice of the basis of the recurrent connection matrices, regular grid fields in 2D and 3D spaces can emerge.

While generating regular grid fields is a basic requirement, the plane dependency of our model further presents biologically plausible interpretations of the experimental observations. First, during the computation, motion can be projected onto the planes. The projection may happen when path integration is on a manifold rather than a volumetric space. As a result, path integration is anisotropic, as observed in Hayman et al. (2011), Hayman et al. (2015), and Casali et al. (2019). Second, the perception of planes could be uncertain. A perceptual error occurs due to the limitation in the sensors, cognitive errors, to name a few. The error could perturb the RNN updates and explain why grid fields lose global order in volumetric navigation reported by Grieves et al. (2021) and Ginosar et al. (2021). Third, the model allows switching among navigation modes categorized by plane definition, grid cell periodicity, perceptual error, etc. This capacity makes our model more biologically plausible than other models that merely work on a single regular arena because, in nature, navigation is a combination of various simple modes (Finkelstein et al., 2016).

We test our model with simulated trajectories in 2D and 3D, and investigate how the uncertain perception of planes influences grid field regularity. Simulated grid fields in hexagonal lattice directly emerge during planar navigation. During multilayered navigation and navigation on manifolds, the projection of motion leads to anisotropic grid fields. Grid fields during volumetric navigation ideally fit FCC, but the uncertain perception of planes diminishes global order. The simulations further predict that the degradation of regularity increases as the time interval between two consecutive perceptions decreases. Finally, we compare our model with two interpretive models considering special 3D cases (Horiuchi and Moss, 2015; Wang et al., 2021) and two training models in 3D space (Stella and Treves, 2015; Soman et al., 2018), and provide suggestions on experiments and possible extensions to our model.

## 2. Method

Path integration maps egocentric motion information to allocentric position information. The egocentric motion information can be represented with 6-degree-of-freedom (6-DoF) motion. In 6-DoF motion, a rigid body rotates and translates about three orthogonal axes with origins at the body, thus necessitating six variables to represent the changes. The two egocentric velocities should undergo a cognitive process before becoming the direct input to grid cells.

The first component, section 2.1, is a model of the possible cognitive representation of the 6-DoF motion. We put forth that the egocentric velocity information is represented with respect to perceived planes. Next, a von Mises-Fisher random process is introduced for the uncertain perception of planes. In section 2.3, we describe and derive a weight-variable RNN model for generating grid cell spiking from the perceived motion. Finally, trajectory generation, prototypical structure generation, and analytical metrics are described in the sections 2.4–2.6.

### 2.1. A Representational Model of 6-DoF Motion in 3D Space

Denote the 3D position of an animal at time *t* by **x**_*t*_. The animal has rotational velocity **ω**_*t*_ and translational velocity **v**_*t*_. Let ‖**ω**_*t*_‖ = θ_*t*_.

For θ ≠ 0, as per Chasles' theorem (Chasles, 1830), the motion is identical to simultaneous rotation about a screw axis and translation along that axis. This is called the screw axis representation (**u**_*t*_, *h*_*t*_, **q**_*t*_).

- **u**_*t*_ = **ω**_*t*_/θ_*t*_ is the unit vector along the screw axis.
- $h_{t}={\text{v}}_{t}\cdot \omega_{t}/\theta_{t}^{2}$ is the screw pitch, i.e., the ratio of the linear speed to the angular speed.
- **q**_*t*_ = **u**_*t*_ × **v**_*t*_/θ_*t*_ is a point on the screw axis, marking the displacement of the axis from the origin.

In this study, we assume that the animal performs egocentric-allocentric transformation (Finkelstein et al., 2016; Bicanski and Burgess, 2020) on either the velocities or the screw axis representation. Either way produces the same allocentric neural encoding of motion based on the screw axis. To avoid ambiguity, in the following content, right superscript *w* stands for vectors in the reference frame with respect to the 3D world or gravity, $F_{t}^{w}$.

**Figure 1:**
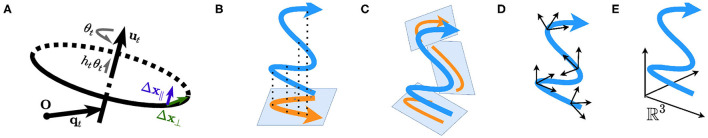
**(A)** A Schematic of the screw axis system. **(B–D)** Plane-dependent computation. **(B)** The motion is projected onto a single plane, such as the horizontal plane defined by gravity and is a special case of **(C)**. **(C)** The motion is projected onto instantaneous planes, such as the tangent planes to the motion manifold. **(D)** Path integration depends on the local cylindrical systems with respect to the planes. **(E)** Plane-independent computation; the path is interpreted as a curve in the static Euclidean space ℝ^3^.

We assume that within a short time window Δ*t*, the animal is performing a uniform helical motion. We are able to decompose the displacement Δ**x** into two components: Δ**x** = Δ**x**_⊥_ + Δ**x**_∥_ (refer to section 1, Supplementary Material). Δ**x**_⊥_ is the displacement on the plane defined by ${\text{u}}_{t}^{w}$ (equivalently, the displacement perpendicular to ${\text{u}}_{t}^{w}$) and Δ**x**_∥_ is the elevation along ${\text{u}}_{t}^{w}$ (the displacement parallel to ${\text{u}}_{t}^{w}$). Figure 1A illustrates the screw axis system and ‖Δ**x**_⊥_‖, ‖Δ**x**_∥_‖.

For convenience, let *r* = ‖Δ**x**_⊥_‖ and *b* = ‖Δ**x**_∥_‖. Define a cylindrical coordinate system with respect to **u**_*t*_: (*r*, ϕ, *b*). Here, ϕ is the direction of planar displacement. The representation of Δ**x** in this coordinate system is given by Equation 1:


(1) $$\begin{array}{l} \Delta x={\hat{M}}^{u}\left(\begin{array}{c} r_{t}\cos\phi_{t}\\ r_{t}\sin\phi_{t}\\ b_{t} \end{array}\right)=r_{t}{\hat{M}}^{u}\left(\begin{array}{c} \cos\phi_{t}\\ \sin\phi_{t}\\ 0 \end{array}\right)+b_{t}{\hat{M}}^{u}\left(\begin{array}{c} 0\\ 0\\ 1 \end{array}\right)\\ \;=r_{t}M^{u}\gamma_{t}+bu_{t}^{w} \end{array}$$

Where, ${\hat{M}}^{u}$ is a rotational mapping ℝ^3^ → ℝ^3^ dependent on ${\text{u}}_{t}^{w}$, *M*^*u*^ is the first two columns of ${\hat{M}}^{u}$, and $\gamma_{t}={\left[\cos\phi_{t},\sin\phi_{t}\right]}^{T}$.

Therefore, a spatial displacement Δ**x** as a result of rotation and translation can be interpreted in three distinct ways:

A1 *b* = 0, *r* ≠ 0
  The animal is performing a circular motion. If the motion is internally represented as 2D, the dimensionality can be reduced by clamping *M*^*u*^ ← *I*. This case is identical to B1 in terms of computation (see below), either as a planar circular motion or a parameterization of a 3D path. This mode could exist in the planar navigation experiments (Hafting et al., 2005; Fyhn et al., 2008; Doeller et al., 2010; Yartsev et al., 2011; Killian et al., 2012; Jacobs et al., 2013; Hayman et al., 2015).
A2 *b* ≠ 0, *r* ≠ 0
  The animal is performing a helical motion. The path can be projected onto the plane defined by ${\text{u}}_{t}^{w}$ and the computation along the vertical axis is degraded or discarded. This mode can explain multilayered navigation: for instance, navigation on a supportive helix (Hayman et al., 2011). The projected path integration is identical to A1 and B1.
A3 *b* ≠ 0, *r* = 0
  The animal is performing a spatial translation with self-spinning, such as a spinning bullet. Currently, there is no strong evidence that clearly demonstrates how self-spinning acts on the entorhinal representation of space. Hence, the computation might be the same as B3 (see below).

Now, consider the case θ = 0, i.e., no rotation. A spatial displacement Δ**x** can also be described by this coordinate system, with three particular interpretations:

1. B1 *b* = 0, *r* ≠ 0; ${\text{u}}_{t}^{w}$ is the normal vector of the plane of navigation
  The animal is performing a planar (2D) translation, e.g., navigating on a surface; *M*^*u*^ ← *I*. This is expected to be in the experiments on horizontal planar navigation (Hafting et al., 2005; Fyhn et al., 2008; Doeller et al., 2010; Yartsev et al., 2011; Killian et al., 2012; Jacobs et al., 2013), slope (Hayman et al., 2015), and vertical wall (Casali et al., 2019).
2. B2 *b* ≠ 0, *r* ≠ 0
  The animal is performing a spatial translation with a reference to a plane, which is real (supportive) or imaginary. The path can be projected onto the plane and the dimensionality is reduced. For example, rats navigating on a vertical pegboard jump vertically most of the time, and the grid fields seem to be projected onto the horizontal plane (Hayman et al., 2011).
3. B3 *b* ≠ 0, *r* = 0; **u**_*t*_ is parallel to the displacement at *t*
  The animal is performing a pure spatial (3D) translation without referring to a plane. This case is divergent from all above in that it does not require perceiving a plane, i.e., plane-independent. It is ergo accurate and does not give rise to plane perception error (refer to section 2.2 for more).

To summarize, we hypothesize that the entorhinal representation of the space during navigation at any time is either anchored on a plane defined by ${\text{u}}_{t}^{w}$ (A1, A2, B1, and B2; Figures 1B–D) or pure 3D (B3; Figure 1E). In cases of A1, A2, B1, and B2, the navigation can be projected onto the instantaneous plane(s) and the dimensionality is thus reduced (Figures 1B,C).

### 2.2. Uncertain Perception of the Changing Axis

The previous section establishes a plane-based representation of motion. The perception of planes should naturally be uncertain, or stochastic: perceptual error could arise from multiple sources, such as the limitation of sensors, illusion, transmission error, and cognitive error. The probabilistic perception is expected to happen in the rotational mapping ${\hat{M}}^{u}$. However, instead of having a joint distribution of the elements of the matrix, stochastic rotational mapping can be modeled by a random variable based on the rotational axis ${\text{u}}_{t}^{w}$. One way to define ${\hat{M}}^{u}$ that satisfies Equation 1 is a rotation of 180° about the axis along the average of ${\text{u}}_{t}^{w}$ and [0, 0, 1]^*T*^. The average is denoted by ${\text{z}}_{t}=\left({\text{u}}_{t}^{w}+{\left[0,0,1\right]}^{T}\right)/2$.


(2) $$\begin{array}{r} {\hat{M}}_{t+\tau }^{u}=2\frac{{\text{z}}_{t}{\text{z}}_{t}^{T}}{{\text{z}}_{t}^{T}{\text{z}}_{t}}-I\text{ for}\frac{t}{\tau }\in {\mathbb{Z}}^{+}\text{(i.e.,}t\text{ is a multiple of }\tau \text{)} \end{array}$$

The derivation is in section 2, Supplementary Material. In this study, we assume that the rotation is discrete-time variable, changing with period τ. Larger τ indicates that less attention, or computation resource, is allocated for such planes. It reduces computation, but may lead to the accumulation of perceptual errors. τ is referred to as “refresh interval” below, because the assumed discrete process is analogous to a monitor that refreshes at a certain rate.

**Figure 2:**
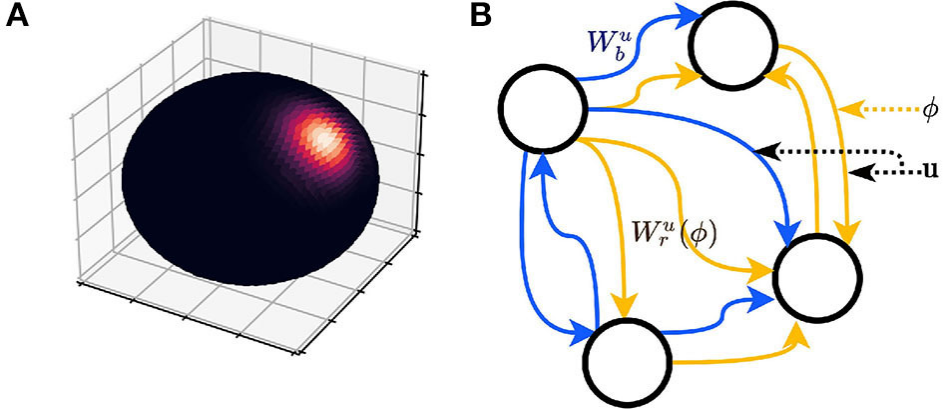
**(A)** Probability density heatmap of a three-dimension (3D) von Mises-Fisher distribution (the axes are arbitrary, so not marked in the figure). The probability is defined on the surface of the sphere only (no probability inside or outside the sphere). The distribution is radially symmetric and centered at the direction defined by **u**. The area of high probability (bright color) is controlled by κ. **(B)** A schematic of the recurrent neural network (RNN) is described by Equations (4, 5). The elements of **a**(**x**) have two pathways, implemented as variable weight matrices $W_{b}^{u}$ and $W_{r}^{u}\left(\phi \right)$.

Now, the deterministic variable ${\text{u}}_{t}^{w}$ is replaced with a continuous random variable **ξ**, i.e., the perceived ${\text{u}}_{t}^{w}$. **ξ** is also a directional vector and follows a 3D von Mises-Fisher distribution, which is a continuous probability distribution defined on the surface of a unit 2-sphere (Fisher, 1953). It is optimal for Gaussian-like distribution of 3D direction vectors. In other words, the probability of perceiving a tilted plane relative to the actual plane is the same for all directions, if the angle between **ξ** and the actual ${\text{u}}_{t}^{w}$ is the same; at the same direction, the probability of perceiving a tilted plane decreases as the angle between **ξ** and the actual ${\text{u}}_{t}^{w}$ increases (Figure 2A). The von Mises-Fisher distribution of a direction **ξ** ∈ ℝ^3^ has the probability density function


(3) $$\begin{array}{r} \begin{array}{c} P\left(\xi |\text{u},\kappa \right)=C_{3}\left(\kappa \right)\exp\left(\kappa \xi^{T}\text{u}\right) \end{array} \end{array}$$

where *C*_3_ is the normalizing factor. **u** anchors the center of the distribution, and the concentration parameter κ controls the width or spread of the distribution. However, different from the variance of a Gaussian distribution, the variation is reversely proportional to κ. Perception is accurate as κ → ∞. Thus, we term κ “perceptual certainty” in the following content.

Finally, for animals performing 2D navigation or navigation projected onto 2D, the third axis could be perceptually neglected, i.e., ${\hat{M}}^{u}\leftarrow I$. The perception of the projection plane is *P*(**ξ**|**u**_0_, κ), where ${\text{u}}_{0}={\left[0,0,1\right]}^{T}$. For animals performing 3D navigation, it is beneficial to have the 3D allocentric reference frame. The perception model is, therefore, $P\left(\xi |{\text{u}}_{t}^{w},\kappa \right)$.

### 2.3. 3D Grid Cell Model Based on a RNN

In the end, there needs a model that generates spikes from motion information. We adopt the model from Gao et al. (2021) and extend it to 6-DoF motion in 3D. Starting from a generic framework, we add more conditions for 3D and derivations for the computation in the vertical direction, and find a biologically meaningful decomposition of recurrent connection matrices. The procedures from which the generic framework is developed are presented in section 3, Supplementary Material. The generic functions in the framework can be implemented with different models, among which the simplest is the linear model. Implementing the framework with linear models results in a RNN, which is presented in this section.

The mEC activities are defined as a vector dependent on its current position, **a**(**x**). Notice that we use the term mEC activities instead of grid cell activities, because each element of **a** does not necessarily stand for a single neuron. It instead could represent a subpopulation of neurons. As we will discuss later, **a** could be the read-out of a few interconnecting ring attractors.

First, we consider an infinitesimal update δ**x**. Let an update on the neural activities be the product between a weight matrix and the original neural activities. The weight matrix is a variable that depends on **u** and ϕ. Moreover, let the weight matrix be the sum of an identity matrix and two variable matrices, $W_{r}^{u}\left(\phi \right)$ and $W_{b}^{u}$. $W_{r}^{u}\left(\phi \right)$ controls the update related to Δ**x**_⊥_, while $W_{b}^{u}$ controls the update related to Δ**x**_∥_. We can write Equation 4 as follows:


(4) $$\begin{array}{r} \text{a}\left(\text{x}+\delta \text{x}\right)=\left(I+W_{r}^{u}\left(\phi \right)\delta r+W_{b}^{u}\delta b\right)\text{a}\left(\text{x}\right) \end{array}$$

The model, with infinitesimal updates, is an RNN, because the elements of **a** form an interconnected graph that meets the descriptions of RNN (Figure 2B). However, this model essentially differs from a typical RNN in that the weight matrix in this study is a dependent variable, while the weights of a typical RNN are constant after parameter tuning.

Because the update is recurrent, the weight matrix is left-multiplied *N* times for a finite displacement Δ**x**. If we let $\delta \text{x}=\underset{N\to \infty }{\lim}\Delta \text{x}/N$, the product of matrices approaches a matrix exponential as *N* → ∞, resulting in another RNN.


(5) $$\begin{array}{l} a(x+\Delta x)=\underset{N\to \infty }{\lim}{\left(I+W_{r}^{u}(\phi )r/N+W_{b}^{u}b/N\right)}^{N}a(x)\\ \;=\exp(W_{r}^{u}(\phi )r+W_{b}^{u}b)a(x) \end{array}$$

The weight matrices $W_{r}^{u}\left(\phi \right)$ and $W_{b}^{u}$ do not require training when they can meet the ideal conditions. That is, the model should be stable given different **u**, ϕ, and *b*, and the neurons should be complimentary for all **x** so as to encode the entire space in scope. We can diagonalize the matrices such that they are linearly based on *M*^*u*^, **γ**_*t*_, and **u**. The derivation is in section 4, Supplementary Material.


(6) $$\begin{array}{l} W_{r}^{u}(\phi )=U\text{diag}(iBM^{u}\gamma_{t})U^{*}\\ \;W_{b}^{u}=U\text{diag}(iBu)U^{*} \end{array}$$

The matrix *B* is the basis, since it represents **u** and $M^{u}\gamma_{t}$ in another set of axes that are encoded in the weights internally. Furthermore, both *B* and *U* can have static solutions that can satisfy the ideal conditions (refer to section 4, Supplementary Material). One of the solutions of *B* and the corresponding *U* is


$$\begin{array}{r} B=b_{0}R_{0}\left(\begin{array}{ccc} 2\sqrt{2}/3 & 0 & -1/3\\ -\sqrt{2}/3 & \sqrt{6}/3 & -1/3\\ -\sqrt{2}/3 & -\sqrt{6}/3 & -1/3\\ 0 & 0 & 1 \end{array}\right), \end{array}$$

(7) $$\begin{array}{r} U=\frac{1}{2}\left(\begin{array}{cccc} 1 & 1 & 1 & 1\\ 1 & \exp\left(-2i\pi /3\right) & \exp\left(i\pi /3\right) & -1\\ 1 & \exp\left(i\pi /3\right) & \exp\left(-2i\pi /3\right) & -1\\ 1 & -1 & -1 & 1 \end{array}\right) \end{array}$$

In this study, *b*_0_ ≠ 0 is a constant finite scale and *R*_0_ is a constant rotation matrix. *b*_0_ and *R*_0_ can be arbitrary. In our simulation, for better visualization, we set *b*_0_ = 10 and *R*_0_ correspond to the rotation of 8 degrees.

The specific solution of the basis *B* in Equation 7 is biologically meaningful in ideal cases. With proper phase differences between neurons, periodic activities along the row vectors of *B* ought to form either HCP or FCC in 3D navigation, as predicted by Mathis et al. (2015). In 2D navigation, periodic activities along the three vectors of its planar projection form the hexagonal lattice (Gao et al., 2021). Preliminary experiments suggest that a good initialization of neural phases is [1, *e*^*iπ*^, 1, *e*^*i*3π/2^]*T*.

Note that **a** is a complex vector. The benefit is that complex values efficiently encode periodicity. On the one hand, each complex element can be considered as the “read-out” of a 1D ring attractor network. The absolute value of a complex element is proportional to the peak of the neural activities in the attractor. The phase of the complex element indicates where the peak locates in the ring attractor. On the other hand, each complex element can be considered as a single neuron whose subthreshold regime is periodic. These neurons are mediated by perception and cognition in a complicated manner.

Ultimately, each element of **a**(**x**) spikes according to a Poisson random process with rate $\lambda ={\left[\lambda_{0}+\exp\left(-c\left(a^{\prime }-\lambda_{1}\right)\right)\right]}^{-1}$ where *a*′ is the normalized real part of an element in **a**. The normalization eases the comparisons to find the appropriate scale *b*_0_. λ_0_, *c*, and λ_1_ are the shape parameters of the logistic mapping. The shape parameters do not affect the results qualitatively as long as the curve of the function remains in an “S” shape and the sharp changing part of the curve is >0.5, i.e., midpoint of the range of *a*′. In all of our simulations, λ_0_ = 1.1, *c* = 15, and λ_1_ = 0.7.

### 2.4. Trajectory Generation

Trajectories are limited in a [−1, 1] × [−1, 1] × [−1, 1] cube. At each time step *t*, Δ**x**_*t,i*_ is first sampled from a uniform distribution *U*(*a*_*i*_, *b*_*i*_), where


(8) $$\begin{array}{r} a_{i}=\max\left\{{\text{x}}_{t-1,i}-\Delta {\text{x}}_{\max,i},-1\right\} \end{array}$$

(9) $$\begin{array}{r} b_{i}=\min\left\{{\text{x}}_{t-1,i}+\Delta {\text{x}}_{\max,i},1\right\} \end{array}$$

For 3D navigation, the algorithm draws ${\text{u}}_{t}^{w}$ from a von Mises-Fisher distribution after every interval τ of refreshing the perception and calculates ϕ_*t*_, *r*_*t*_, and *b*_*t*_ accordingly. For 2D navigation, the altitudes (Z-axis values) are simply discarded. For navigation on manifolds, the altitudes are replaced by the outputs of the functions that describe the manifolds. The trajectories for the results are generated with constant parameters Δ**x**_max,*i*_ = 0.08, κ_*traj*_ = 200 (3D only), and total number of steps *T* = 10^5^.

### 2.5. Prototypical Structure Generation

For FCC and HCP structures, the vertices that fall into the [−1, 1] × [−1, 1] × [−1, 1] cube are located. A comparative amount (≈100) of RND vertices is drawn uniformly from the cube afterward. For COL structure, layers of a hexagonal lattice with the same offset are stacked with 1/10 inter-layer distance of FCC or HCP. All the vertices are rotated by 8 degrees along the vertical axis, to be aligned with the simulation. For every vertex of a prototypical structure, 500 points are generated from a 3D normal distribution centered at that vertex. We confirm both visually and *via* MeanShift clustering algorithm that the generated FCC, HCP, and RND have well-separable spherical clusters, while COL has columns.

### 2.6. Metrics to Analyze the Spatial Distributions of Spikes

To date, no single measure perfectly describes the spatial distribution of spike locations from all aspects. We use an array of measures to maximize the comprehensiveness of our analysis on the distribution, involving sparsity index, spatial information, inter-field distance (IFD), structure scores, and modified radial autocorrelation (MRA). Spatial information and sparsity are used widely to describe the regularity of spikes in space. IFD, structure scores, and MRA are adapted to compare the 3D spatial distributions specifically. IFD is based on the spike locations; spatial information and sparsity index are based on the 3D spike distribution of each neuron in each trial; and structure scores and MRA are based on the discrete autocorrelation of the 3D spike distribution.

#### 2.6.1. Sparsity Index and Spatial Information

The spatial information and sparsity index measures the bit per spike and compactness of the firing fields (Skaggs et al., 1993; Jung et al., 1994). Specifically,


(10) $$\begin{array}{l} \text{Spatial Info}.=\sum_{i}p_{i}\frac{\lambda_{i}}{\langle \lambda \rangle }\log_{2}\frac{\lambda_{i}}{\langle \lambda \rangle }\\ \text{Sparsity Index}=\frac{{\langle \lambda \rangle }^{2}}{\langle \lambda^{2}\rangle } \end{array}$$

where λ_*i*_ is the firing rate in bin *i* and *p*_*i*_ is the probability of animal location in bin *i*.

In addition to the typical calculation of the two metrics, we perform standardization similar to Grieves et al. (2021). First, the spatial information and sparsity index of the original spike train of each neuron in each trial is achieved. Then, the means and SDs of spatial information and sparsity index are measured from 50 random shuffles of the original spike train. Using the means and SDs of the shuffled spike trains, the Z-scored spatial information and sparsity index of the trial can be achieved.

#### 2.6.2. Inter-field Distance

Given the spike locations, we apply the MeanShift clustering algorithm (Cheng, 1995) provided by the *scikit-learn* package (Pedregosa et al., 2011) to identify clusters. The bandwidth is 0.25, and the minimal bin frequency is 25. To reject noise, the cluster_all tag is set to false and clusters with sizes smaller than 30 are removed. Lastly, there are fields that only have small portions inside the cube, so the means of those portions are not the real centers. We tentatively eliminate the clusters with means that are too close to the boundaries (distance < 0.05). The IFD are finally calculated from the remaining clusters.

#### 2.6.3. Structure Scores

We follow the method specified by Stella and Treves (2015) and Grieves et al. (2021) to perform planar symmetry analysis on the prototypical structures and adjust the HCP, FCC, and COL scores (χ_*HCP*_, χ_*FCC*_, and χ_*COL*_, respectively) based on the heatmaps of hexagonal grid scores and squared grid scores (Supplementary Figure S1A). Each pixel of the heatmap is the grid score of an oblique slice of the 3D autocorrelation. To calculate the structure scores, first, the local maxima of hexagonal grid score maps, {_α_*i*_}*i*_, and of squared grid score maps, {_β_*i*_}*i*_, are located for HCP, FCC, and COL (Supplementary Figure S1A); then, χ_*FCC*_, χ_*HCP*_, and χ_*COL*_ scores equal median{_α_*i*_}*i*_ + median{_β_*i*_}*i*_. χ_*FCC*_, χ_*HCP*_, and χ_*COL*_ of their structures are significantly higher than of the others; χ_*FCC*_ and χ_*HCP*_ of COL are significantly lower (Supplementary Figure S1B). The averaged scores of their corresponding prototypes will be used for comparisons in the analysis of simulation results.

#### 2.6.4. Modified Radial Autocorrelation

Radially averaged autocorrelation indicates the presence of repeated radial-symmetric patterns. Yet the number of visited bins at a specific radius, *N*(*r*), grows quadratically with the radius, vanishing the mean of autocorrelation especially when the patterns are widely spaced. Hence, to better reveal the spatial patterns, the radial sum of autocorrelation is divided by $\sqrt{N\left(r\right)}$ instead of *N*(*r*). That is, the modified autocorrelation is calculated as $Â\left(r\right)=\frac{1}{\sqrt{N\left(r\right)}}\underset{\sqrt{i^{2}+j^{2}+k^{2}}\in \left(r-1,r\right]}{\sum }A_{ijk}$, where *A* is the autocorrelation and max*r* is the half of the edge length of the cube *A*.

## 3. Results

Experiments are done to verify and investigate the following:

1. Our model is capable of generating regular grid fields during different navigation modes (section 3.1).
  This is the foundation of our hypothesis that perceptual error degrades grid field regularity (investigated in 2) and compatibility with mode switching (investigated in 3). We test the model with trials on planar, multilayered, manifold, and volumetric navigation.
2. Uncertain perception reduces grid field regularity, especially in volumetric navigation where grid fields lose global order and only preserve local order (sections 3.2, 3.3).
  Our simulations support this hypothesis. We further analyze how the grid field regularity is manipulated by two perceptual parameters: perceptual certainty, κ, and refresh interval, τ (refer to section 2.2). We found that decreasing refresh interval gradually deprives the global order of grid fields. κ, on the other hand, has a non-linear influence on the grid field regularity.
3. The model allows switching among modes (section 3.4).
  We simulate the trajectories in a half-flat, half-tilted arena to demonstrate compatibility of our model with mode switching. Mode switching is stable as long as a neural phase restoring mechanism exists.

### 3.1. Numerical Simulation of the Grid Field

We numerically simulated enough trials of grid cell spiking with different perceptual certainty κ and refresh interval τ. Eight trajectories of 10^5^ time steps are first generated, evenly covering the [−1, 1] × [−1, 1] × [−1, 1] cube multiple times. For each trajectory, the plane-dependent modes (A1, A2, B1, and B2) are run with *b*_0_ = 10, κ ∈ {300, 400, 500, 600, ∞}, and τ ∈ {1, 5, 10, 50, 10^2^, 5 × 10^2^, 10^3^, 5 × 10^3^, 10^4^, 5 × 10^4^, 10^5^}. The four neurons of the same network share similar characteristics, but have different phases. This has a minor effect on the following analyses, all of which are shift-invariant. Hence, we achieve 32 samples for each (κ, τ).

**Figure 3:**
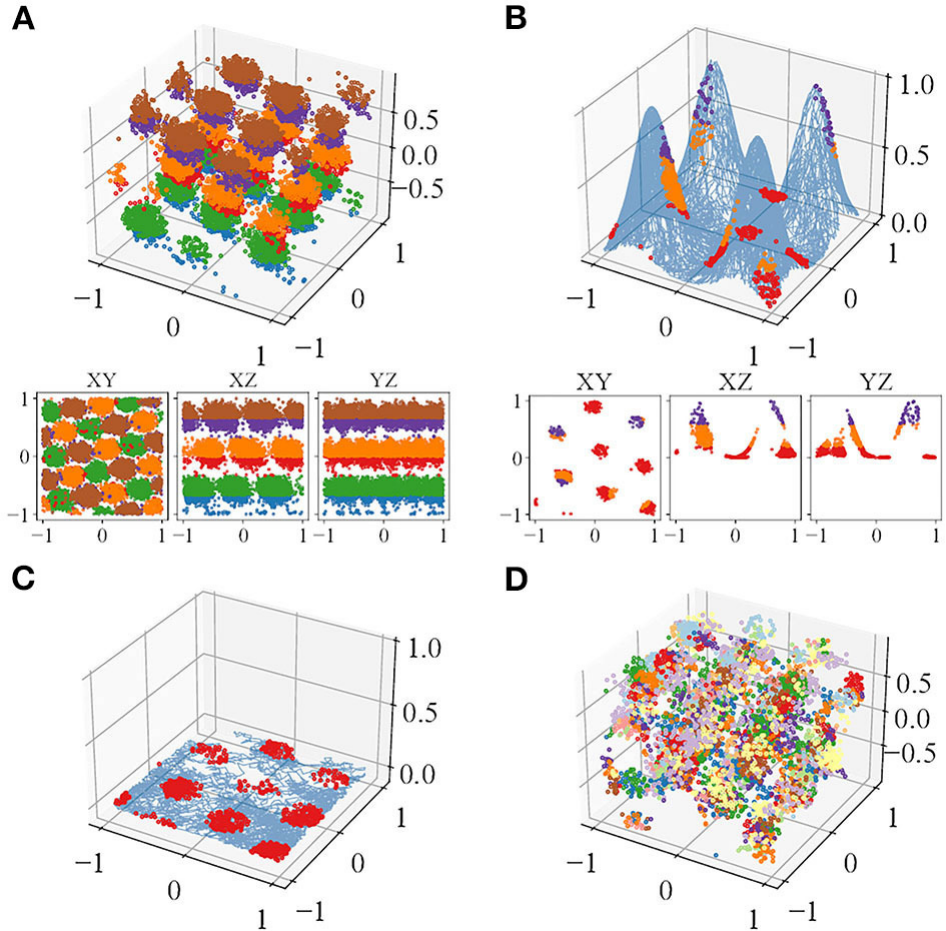
An example of the spike locations in trials with accurate perception, colored by Z-axis intervals, of one of the neurons during **(A)** volumetric navigation, **(B)** navigation on a manifold formed from four Gaussian surfaces, and **(C)** Two-dimensional (2D) navigation or navigation projected onto the horizontal plane. **(D)** Trial with perceptual uncertainty, colored by the refresh intervals. The blue curve in **(B,C)** show the trajectories.

Visually, the locations of the simulated spikes agree with our theoretical prediction. For plane-dependent modes, spike locations of trials with both accurate (κ → ∞) and uncertain perception form clusters (Figure 3). During volumetric navigation, the structures of the trials with accurate perception resemble FCC structure from observation (Figure 3A), while those of trials with perceptual uncertainty are not visually identifiable (Figure 3D). Planar navigation shows hexagonal lattice (Figure 3C), confirming with the experimental observations (Hafting et al., 2005; Fyhn et al., 2008; Doeller et al., 2010; Yartsev et al., 2011; Killian et al., 2012; Jacobs et al., 2013). Navigation on some simple manifolds is simulated as well with τ = 10 and case B1 (Figure 3B), and the grid fields are similar to those predicted by Wang et al. (2021). The grid fields form hexagonal lattices when projected onto the horizontal plane. The plane-independent mode (section 2.1, B3) only has trials with accurate perception since it does not incur plane perception error. The trials with accurate perception have little difference compared with those of the plane-dependent modes in terms of any metrics used.

### 3.2. Grid Fields Gradually Lose Global but Not Local Order as Refresh Interval Decreases

**Figure 4:**
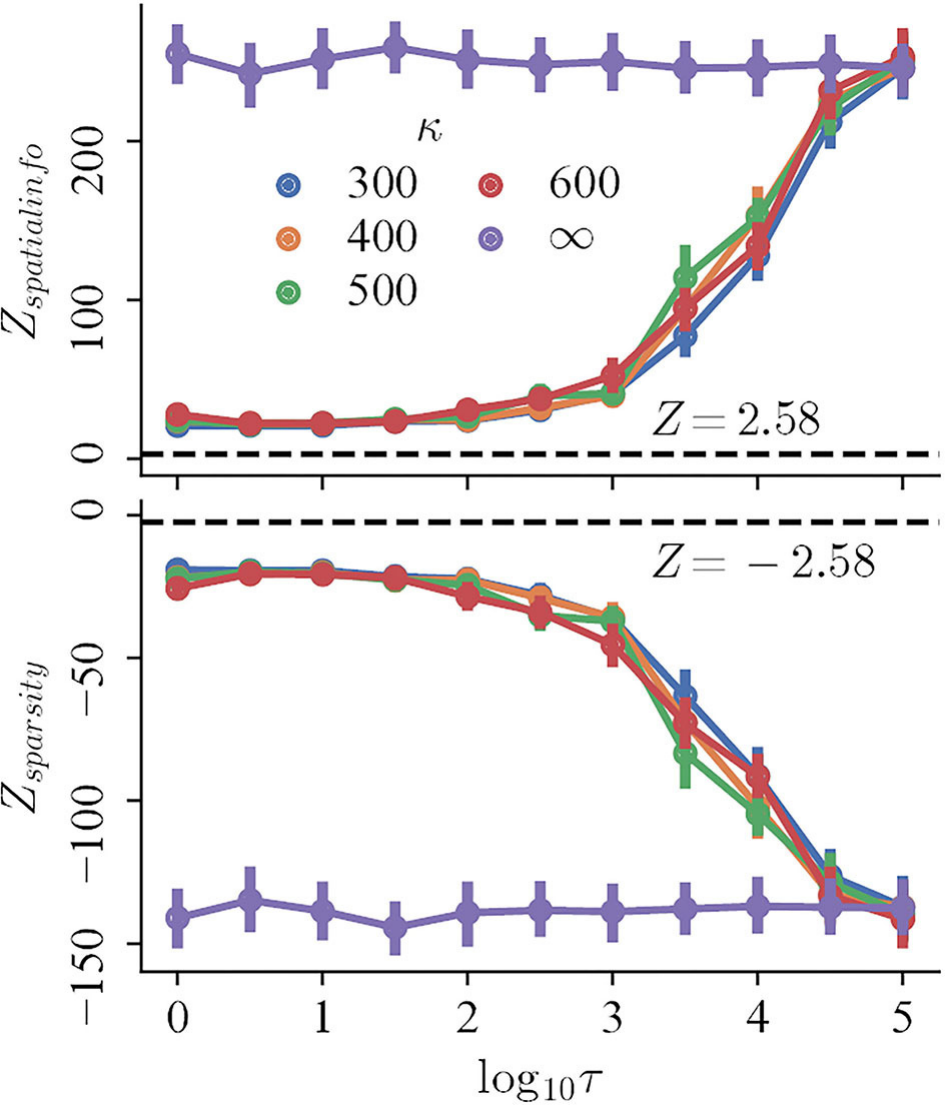
The Z-scored spatial information (top) and Z-scored sparsity index (bottom) of trials with variable refresh interval τ and perceptual certainty κ. Black dashed lines mark *Z* = 2.58.

To begin with, the regularity, measured by the Z-scored spatial information and sparsity index, changes monotonically with decreasing refresh interval for trials with perceptual uncertainty, but not trials with accurate perception. The mean Z-scored spatial information (sparsity index) of the trials with perceptual uncertainty of different κ ≪ ∞ decreases (increases) rapidly for τ ∈ {10^3^, 5 × 10^3^, 10^4^, 5 × 10^4^}, and converges for lower τ. In contrast, both the spatial information and sparsity index of trials with accurate perception remains high and nearly constant for all τ (Figure 4, purple curves; Pearson correlation, *r* = −0.042 and *r* = 0.031, respectively). Finally, the magnitudes of Z-scores of all trials are significantly larger than 2.58, indicating that they are more compact and carry more spatial information than those random shuffles.

After getting a sense of the regularity of the spatial distribution of spikes, we measure how similar the distributions are to the prototypes, given different τ and κ. There does not exist a single measure that perfectly describes the similarity, so multiple methods are used, including IFD distribution, structure scores, and MRA.

**Figure 5:**
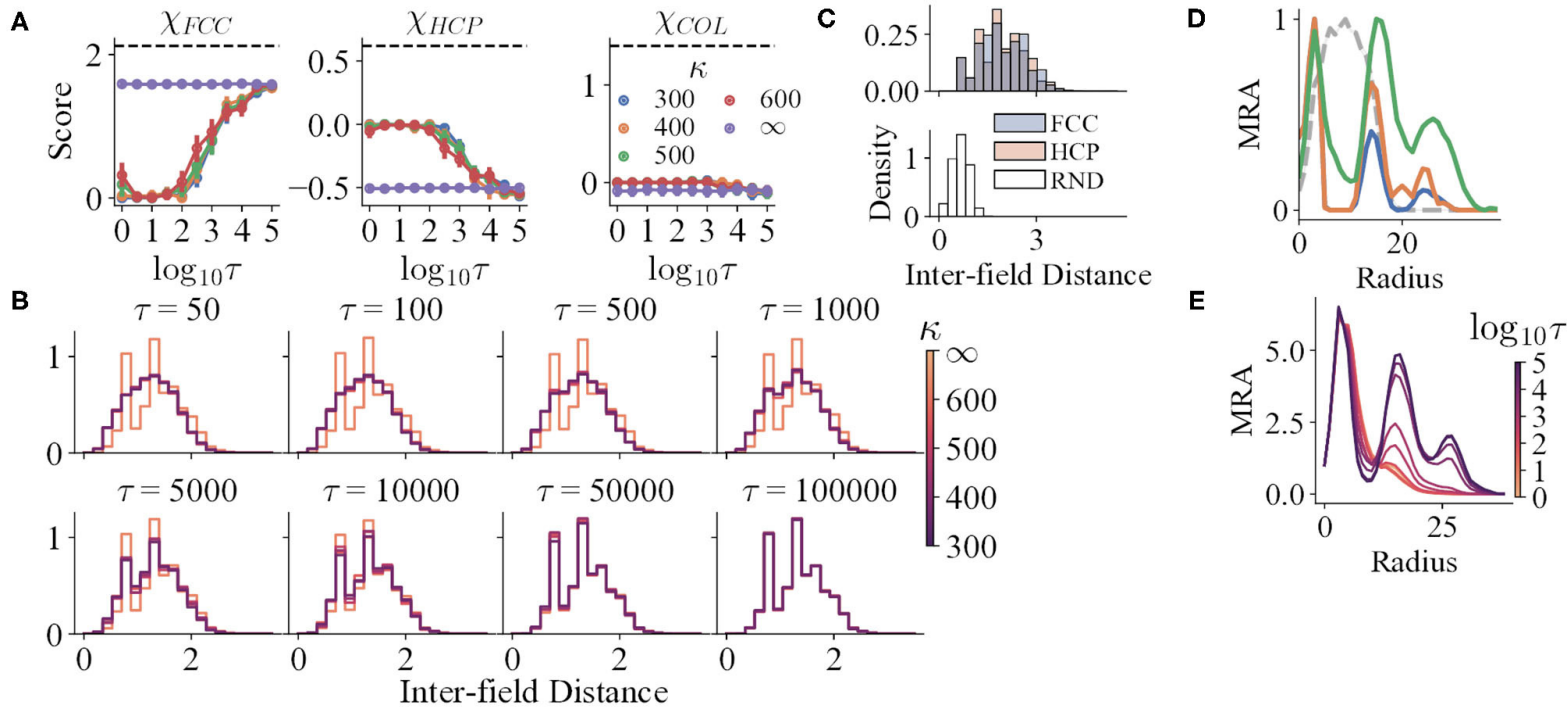
**(A)** The structure scores of the simulation results with variables τ and κ. The black dashed lines indicate the mean scores of the corresponding prototypes. **(B,C)** The IFD distributions of the simulation results and prototypes, respectively. The distributions for τ ∈ {1, 5, 10} are not shown, for they are almost the same as that of τ = 50. **(D)** The mean curves of the MRA of RND (grey dashed line), HCP (blue), FCC (orange), and COL (green). **(E)** The mean curves of the MRA of the simulation results, respectively.

Trials with accurate perception show obvious global order. The structure scores (Figure 5A), the spike locations of the trials with accurate perception (Figure 3A), and the shapes of the mean MRA (Figures 5D,E) together manifest that the ideal grid fields of the model fit into FCC. All the three structure scores of the trials with accurate perception for all τ remains nearly constant (Pearson correlation, *r*_*FCC*_ = −0.007, *r*_*HCP*_ = 0.023, and *r*_*COL*_ = 0.006), whereas χ_*FCC*_ remains significantly higher and close to the ideal value.

For trials with perceptual uncertainty, as the refresh interval decreases, χ_*FCC*_ decreases in general, indicating that the field structures gradually deviate from FCC with decreasing τ (Figure 5A). Nevertheless, though the corresponding χ_*HCP*_ and χ_*COL*_ increase, their maxima are still qualitatively lower than the reference values (black dashed lines). The shapes of the mean MRA curves also differ from that of HCP or COL. Thus, as τ changes, the structures resemble neither HCP nor COL. The gradual change of IFD distribution shape when τ reduces also reinforces the argument (Figure 5B).

Trials with lower τ still preserve local order to some degree. In addition to the analysis that all trials have significantly higher spatial information and sparsity index than random shuffles, both the IFD distribution and MRA of the RND prototype reject that the fields are completely randomly distributed. Specifically, the IFD distribution of RND is unimodal, narrow, and right-skewed (Figure 5C), for RND fields are allowed to be much closer. Yet, the distributions of the trials with small τ are wider and less skewed, possibly as an outcome of a higher minimal IFD. Furthermore, the MRA of the simulated fields still maintains a small second peak or a shoulder (Figure 5E). Hence, weak repeats of radially symmetric patterns could exist locally.

### 3.3. The Perceptual Certainty Affects the Structure in a Complex, Subtle Manner

Spatial information (sparsity index) and κ seem to have a non-linear relationship. Trials with perceptual uncertainty with all κ have no significant differences when τ ∈ {5, 10, 50, 5 × 10^4^, 10^5^} (*t*-tests, *p* > 0.05). With the other τ values, at least one of the distributions corresponding to κ ≪ ∞ significantly differs from one of the others (*t*-tests, *p* < 0.05). However, these distribution means do not manifest a constant sequence as τ changes, indicating a non-linear mapping. Similar observations exist in structure scores and MRA.

Moreover, κ does not interfere with the progress of losing global order. For κ ≪ ∞, the IFD distributions do not differ qualitatively at a certain τ (Figure 5D). Similarly, the MRA always loses the farther peak first as τ decreases. The locations of the three peaks in the MRA do not change with κ as well, even though the peak heights vary (Figure 5E).

Together, the data purports that κ has an indirect effect on the field locations. It might alter the internal density of a firing field, affecting MRA and structure scores, both of which are sensitive to the internal density. Change in local spike density might influence spatial information and sparsity density in a subtle manner as well. Further analysis of the effect of κ is out of interest in this study.

### 3.4. Mode Switching

**Figure 6:**
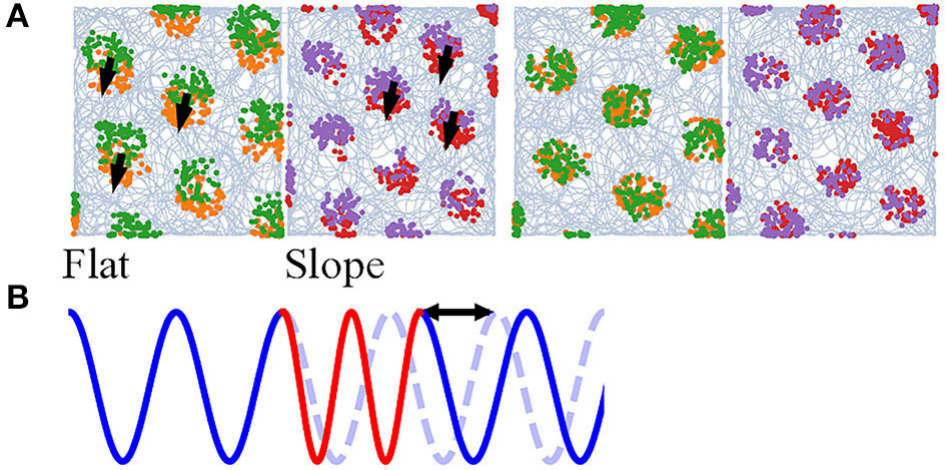
**(A)** Simulation of an animal starting on a flat region (orange), entering the slope for the first time (red), returning (green), and entering the slope again (purple). Left: without phase correction, the firing pattern is translated after return to either side. Right: with phase correction at the time of re-entering either of the regions, the new pattern has the same phase as the old one. **(B)** A 1D illustration of the occurrence of a phase shift due to mode switching. Blue and red stand for two modes and the blue dashed line for the original phase.

Navigation on a half-flat, half-tilted arena is simulated to demonstrate how our model enables mode switching between two navigation modes. Navigating on a horizontal plane (Figure 6A, Flat) gives rise to grid codes that contain all the information, since the altitude is constant, whereas navigating on a tilted plane (Figure 6A, Slope) discards the altitudes due to vector projection. Furthermore, the grid scales are unequal to represent the effects of other sensory cues. The animal visits the arena in the order: Flat, Slope, Flat, Slope (Figure 6A, orange, red, green, and purple, respectively). The time navigating in each plane is similar, though not necessarily the same, and larger than 5,000 steps. If the alternation between the planes is too fast, without a phase restoring mechanism (shown below), rapid alternation in *b*_0_ will deteriorate the stability of neurons.

Mode switching does not deteriorate the computation. The model is able to form hexagonal lattices during the first and second visits. However, the grid fields of the second run on either of the plane are translated (Figure 6A, left). If the frequency of mode switching is high, the grid fields are dispelled (visualization not shown). The spatial translation is found to be the result of phase shift in the neurons; a 1D illustration is in Figure 6B. The phase shift might impede path integration and stability of the cognitive map, so there needs a correction mechanism.

We hypothesize that memory and other somatosensory cues provide the reset current that eliminates the phase shift. In this study, we devise a very simple algorithm: when the animal enters either of the planes for the second time, the activity pattern of the neurons at the previous exit is recovered (memory retrieval). After that, it achieves the displacement between the current position and exit position (interaction between memory and somatosensory cues to estimate the displacement). 100 updates, as if the animal navigates from the first exit position to the current position, are done to restore the previous phase (recognition). The grid fields of the second run overlap well with the first when such “virtual walk” exists (Figure 6A, right).

## 4. Discussion

We construct an interpretive plane-dependent model of 3D grid cells. The model has two achievements: it is capable of representing both 2D and 3D space during different navigation modes and predicting the principles behind the multimodality of grid codes and degradation of grid field regularity observed in experiments (Hayman et al., 2015; Casali et al., 2019; Ginosar et al., 2021; Grieves et al., 2021). Specifically, the model interprets 3D 6-DoF motion in local cylindrical coordinate systems based on planes. The planes can be instantaneous, such as the tangent planes of motion manifolds, or static, such as the horizontal plane defined by gravity. The perceptual error of the planes gives rise to the degradation of grid field regularity from optimal structure. This idea is verified by a weight-variable RNN model extended from (Gao et al., 2021). Furthermore, simulations demonstrate that as refresh interval τ decreases, the degradation gets stronger, but converges at a level better than random placements. Finally, the model naturally bears switching between modes. We give an example with a half-flat, half-tilted arena that involves switching between two modes. The example tells that there has to be a phase restoring mechanism, which is hypothesized to be the interaction between memory and somatosensory cues. We put forward a “virtual walk” algorithm to restore the phase successfully, such that the grid fields are stable on revisiting.

Plane-independent computation (section 2.1, B3) is supported by our model as well but discouraged for two reasons. First, it does not fully agree with the experiments. In this mode, the motion is evaluated under the global Cartesian coordinate system. Merely FCC grid fields can occur, which has not been discovered experimentally. This study in contradiction with (Hayman et al., 2015; Casali et al., 2019) may even arise because the motion planes do not intersect FCC with a few specific angles that lead to hexagonal lattice. The mode does not explain the grid fields during volumetric navigation (Ginosar et al., 2021; Grieves et al., 2021) either. The contradictions may only be solved if a remapping mechanism exists (Finkelstein et al., 2016). Second, this mode is highly subject to motion detection error: little perturbation to the motion directions can drastically eliminate the grid fields by dispersing the spikes. The plane-dependent modes are instead more tolerable to motion detection error. To sum up, plane-dependent computation is more biologically plausible and stable than plane-independent computation.

Our unified framework has some connections to the two interpretive models that deal with only some special cases in 3D: Wang et al. (2021) on crawling on curvy surfaces (2.5D) and Horiuchi and Moss (2015) on 3D free motion. The former establishes a plane-dependent system as well. The major difference is in the cognitive process. Our theory supposes that animals perceive motion in a local cylindrical system and perform a projection, while theirs has motion in a global coordinate system and rotates egocentric basis according to the current tangent plane of the crawling manifold. Since only dealing with crawling, the model has merely 3 co-planar basis vectors which are equivalent to the projection of *B* along the Z-axis. This implementation naturally limits their model on crawling only. The latter addresses 3D free motion. It uses four ring attractors corresponding to four basis vectors identical to our *B*. Each grid cell reads out the logical AND combination of four nodes from the four attractors. In terms of representation, our four complex-valued nodes can be thought of as the simplification of the four attractors. However, their attractors can only form stripes, whereas our recurrent coupling enables each node to form grid fields. This study, nevertheless, does not account for the grid fields on slopes and vertical walls (Hayman et al., 2015; Casali et al., 2019) and the loss of global order (Ginosar et al., 2021; Grieves et al., 2021).

Two other theoretical studies approach the question *via* synaptic plasticity (Stella and Treves, 2015; Soman et al., 2018). Grid fields either arise from mature, gaussian-like place fields (Stella and Treves, 2015) or as a combination of effects from Hebbian plasticity with the integrated head-direction input and anti-Hebbian plasticity in the recurrent connections (Soman et al., 2018). In accordance with our study, they both conclude that grid fields have the tendency to form FCC structure, but may only preserve local order in reality. However, the two plasticity-based models argue the imperfection stems from insufficient training, a lack of either training time (Stella and Treves, 2015) or narrow pitch distribution (Soman et al., 2018). The training time hypothesis may contradict the fact that decay of grid fields regularity is present in model animals raised in enriched 3D environments (Casali et al., 2019; Grieves et al., 2021). Moreover, many environments, especially rectangular ones, share many similarities. Cumulative learning is possible. In addition, replay in hippocampus (Wilson and McNaughton, 1994; Skaggs and McNaughton, 1996; Foster, 2017) and mEC (Ólafsdóttir et al., 2016; Gardner et al., 2019; Trettel et al., 2019) might reinforce the training as well (Bellmund et al., 2018). As such, the training time hypothesis could be rejected. On the other hand, novel stimuli activate the neuromodulatory system that affects synaptic plasticity and reinforcement learning (Schultz, 2002; Ranganath and Rainer, 2003; Nyberg, 2005), questioning the narrow pitch distribution hypothesis. Although animals naturally have narrow pitch distribution, the perception of pitch is intact (assumed by the model and at least present in bats Finkelstein et al., 2016). It is still possible that the learning efficacy is greater for unfamiliar pitch directions than familiar ones, which leads to nearly optimal 3D grid fields. In conclusion, training issues can be overcome by neural adaptation and are less likely.

More experiments on the grid fields with different navigation modes are also necessary to verify our theory. Besides, it is still unclear if the plane-based system exists. This can be broken down into multiple subtasks. People need to confirm the neural basis of (1) the decomposition Δ**x**_⊥_ and Δ**x**_∥_, (2) the cylindrical coordinate system (essentially the perception of **u**), (3) the basis vectors *B*, and (4) variable weights in favor of the derivation above. The existence of gravity-tuned neurons (Laurens et al., 2016) supports the vertical unit vector of *B*, and may further allow such a basis to evolve. For (4), the neural circuits might implement the matrix exponential $\exp\left(W_{r}^{u}\left(\phi \right)r+W_{b}^{u}b\right)$ with a Taylor approximation of finite order. In addition, from a functionalistic perspective, does a “plane” present in cognition? Experimenters might test this by introducing illusions. For example, one can design an optical illusion with checkers. Another possible design is to let animals wear shoes that deceive the accurate proprioception. Distortion to the grid fields may indicate that planes exist.

Finally, multiple theoretical extensions of the model can be made. A spiking neural network (SNN) are more biologically plausible than ours. Till this paper is written, there is no SNN models of grid cell computation beyond horizontal planar navigation. As discussed above, the complex-valued model has connections to ring attractors, so an equivalent network should be feasible. The recurrent connection matrix can be determined *via* convex optimization on objective functions such as Gao et al. (2021), and then the RNN is transformed to SNN. Alternatively, an SNN could be achieved through unsupervised learning directly. Another important direction is to delve into navigation mode switching. The nervous system may not implement the “virtual walk” algorithm applied here to restore the phase. Instead, the phase could be restored directly in an associative memory network, in which reasoning modules provide the restoring currents. Third, an egocentric-allocentric transformation is presumed in this study, but a specific implementation is out of scope. It would be interesting to devise a model for this mapping in a global or 3D point of view. Finally, this study attempts to recover and explain the grid field structures in various navigation modes but does not address decoding. How does the degradation of regularity influence decoding? Previous decoding algorithms developed on planar hexagonal grid fields (Bush et al., 2015; Stemmler et al., 2015; Yoo and Vishwanath, 2015; Yoo and Kim, 2017) are yet to be tested with non-planar modes. For the scenarios that include projection, for sure they are unable to recover the global positions of animals; are they capable of returning the parametric positions on the manifold? If not, new generalizable decoding algorithms should be brought forward. With greater complexity and biological plausibility, we wish our framework can stimulate modeling and experimental efforts to elucidate the computation of grid cells.

## Data Availability Statement

The datasets presented in this study can be found in online repositories. The names of the repository/repositories and accession number(s) can be found below: https://github.com/gongziyida/GridCells3D.

## Author Contributions

ZG and FY conceived the goal and cardinal ideas of the study and drafted the manuscript. ZG derived mathematical equations, implemented and ran the simulations, and analyzed the simulation results. Both authors contributed to the article and approved the submitted version.

## Funding

This work was partly supported by the National Nature Science Foundation of China (Nos. 62088102, 61836004), National Key R&D Program of China 2018YFE0200200, Brain-Science Special Program of Beijing under Grant Nos. Z181100001518006, Z191100007519009, the Suzhou-Tsinghua innovation leading program 2016SZ0102, and CETC Haikang Group-Brain Inspired Computing Joint Research Center.

## Conflict of Interest

The authors declare that the research was conducted in the absence of any commercial or financial relationships that could be construed as a potential conflict of interest.

## Publisher's Note

All claims expressed in this article are solely those of the authors and do not necessarily represent those of their affiliated organizations, or those of the publisher, the editors and the reviewers. Any product that may be evaluated in this article, or claim that may be made by its manufacturer, is not guaranteed or endorsed by the publisher.
