# Supplementary material for: A Plane-Dependent Model of 3D Grid Cells for Representing Both 2D and 3D Spaces Under Various Navigation Modes
Source: Front Comput Neurosci. 2021 Sep 22;15:739515. doi: 10.3389/fncom.2021.739515 (PMC8493087; doi:10.3389/fncom.2021.739515)
Supplement: Supplementary file 1 [file Data_Sheet_1.PDF]

Supplementary Material

1 TWO COMPONENTS OF THE DISPLACEMENT UNDER THE UNIFORM HELICAL MOTION ASSUMPTION

Under the uniform helical motion assumption, in the small time window Δt, the angular velocity, the magnitude of the velocity perpendicular to the rotation axis, and the velocity parallel to the rotation axis $\mathbf{u}_t$ are all constant. It is then easy to express $\Delta \mathbf{x}_\perp$ and $\Delta \mathbf{x}_{||}$ with the screw axis parameters.

$$\begin{aligned} \Delta \mathbf{x}_\perp &= \mathbf{q}_t^w - R(\theta_t \Delta t)\mathbf{q}_t^w \\ \Delta \mathbf{x}_{||} &= h_t \mathbf{u}_t^w \theta_t \Delta t \end{aligned} \tag{S1}$$

where $R(\theta_t \Delta t)$ is a rotational mapping with angle $\theta_t \Delta t$. This rotational matrix is not directly known in the content. However, this information can be provided by the change in the activities of 3D head direction cells, which encode head direction information in 3D space (Finkelstein et al., 2015, 2016).

2 ROTATIONAL MAPPING FOR THE UNCERTAIN PERCEPTION

Noise cannot be easily added to the elements of $\hat{M}^u$ to establish a stochastic rotational mapping, because the time-dependent rotational matrix $\hat{M}^u$ is an element of the Lie group $\mathcal{SO}(3)$, where addition is not defined. Nevertheless, addition is defined in the corresponding Lie algebra $so(3)$. Furthermore, $so(3)$ is the space of the skew-symmetric matrix corresponding to the unit axis of rotation, $\mathbf{v}$, and rotational angle, θ. Hence, it is simpler to work on $so(3)$. It is a common practice to define the operation $[\cdot]_\times$ that maps a vector in $\mathbb{R}^3$ to the skew-symmetric matrix space:

$$\left[\theta \begin{pmatrix} v_1 \\ v_2 \\ v_3 \end{pmatrix}\right]_\times = \theta \left[\begin{pmatrix} v_1 \\ v_2 \\ v_3 \end{pmatrix}\right]_\times = \theta \begin{pmatrix} 0 & -v_3 & v_2 \\ v_3 & 0 & -v_1 \\ -v_2 & v_1 & 0 \end{pmatrix} \tag{S2}$$

Then, we are able to express the rotation using the exponential mapping from $so(3)$ to $\mathcal{SO}(3)$.

$$\hat{M}^u_{t+\tau} = \exp\left(\pi \left[\frac{\mathbf{z}_t}{\|\mathbf{z}_t\|}\right]_\times\right) \tag{S3}$$

For convenience, let $Z = \left[\frac{\mathbf{z}_t}{\|\mathbf{z}_t\|}\right]_\times$. Skew-symmetric matrix Z has the properties:

1. $Z^2 = \mathbf{z}_t \mathbf{z}_t^T - I$
2. $Z^3 = -Z$

We are able to apply the properties in the Taylor series of the matrix exponential to achieve an efficient calculation of the rotation matrix:

$$\begin{aligned}
\hat{M}_{t+\tau}^{u} &= \exp\left(\pi Z\right) \\
&= \sum_{n=0}^{\infty} \frac{\pi^n Z^n}{n!} \\
&= I + \pi Z + \frac{\pi^2 Z^2}{2} + \frac{\pi^3 Z^3}{3!} + \frac{\pi^4 Z^4}{4!} + \frac{\pi^5 Z^5}{5!} + \frac{\pi^6 Z^6}{6!} + ... \\
&= I + \pi Z + \frac{\pi^2 Z^2}{2} - \frac{\pi^3 Z}{3!} - \frac{\pi^4 Z^2}{4!} + \frac{\pi^5 Z}{5!} + \frac{\pi^6 Z^2}{6!} + ... \\
&= I + Z \sum_{n=0}^{\infty} \frac{(-1)^{2n+1} \pi^{2n+1}}{(2n+1)!} + Z^2 - Z^2 \sum_{n=0}^{\infty} \frac{(-1)^{2n} \pi^{2n}}{(2n)!} \\
&= I + Z \sin\pi + Z^2 - Z^2 \cos\pi \\
&= I + ZZ^T - I + ZZ^T - I \\
&= 2\frac{\mathbf{z}_t \mathbf{z}_t^T}{\mathbf{z}_t^T \mathbf{z}_t} - I
\end{aligned} \tag{S4}$$

3 A GENERIC FRAMEWORK OF THE 3D GRID CELL MODEL

Like (Gao et al., 2021), for every move, we suppose there exists a transfer function of the neural activities $\mathbf{a}$ and the displacement $\Delta\mathbf{x}$. It follows that a transfer function of $\mathbf{a}$, r, b, ϕ, and $\mathbf{u}$ should also exist.

$$\mathbf{a}(\mathbf{x} + \Delta\mathbf{x}) = F(\mathbf{a}(\mathbf{x}), r, b, \phi, \mathbf{u}) \tag{S5}$$

The transfer function should work for arbitrary $\Delta\mathbf{x}$. That means if $\Delta\mathbf{x} = \Delta\mathbf{x}_1 + \Delta\mathbf{x}_2$,

$$\mathbf{a}(\mathbf{x} + \Delta\mathbf{x}_1 + \Delta\mathbf{x}_2) = F[F(\mathbf{a}(\mathbf{x}), r_1, b_1, \phi_1, \mathbf{u}_1), r_2, b_2, \phi_2, \mathbf{u}_2] \tag{S6}$$

Consider an infinitesimal displacement δx. With Taylor series approximation, an update on the neural activities $\mathbf{a}(\mathbf{x} + \delta\mathbf{x})$ can be represented as the original activities plus two small drives that depend on the two components of displacement, δr and δb, separately.

$$\begin{aligned}
\mathbf{a}(\mathbf{x} + \delta\mathbf{x}) &= F(\mathbf{a}(\mathbf{x}), \delta r, \delta b, \phi, \mathbf{u}) \\
&\approx \mathbf{a}(\mathbf{x}) + (\delta r \frac{\partial}{\partial \delta r} + \delta b \frac{\partial}{\partial \delta b}) F \mid_{\delta r = 0, \delta b = 0} \\
&= \mathbf{a}(\mathbf{x}) + \delta r f_r(\mathbf{a}(\mathbf{x}), \phi, \mathbf{u}) + \delta b f_b(\mathbf{a}(\mathbf{x}), \mathbf{u})
\end{aligned} \tag{S7}$$

There could be a variety of choices for the generic functions f_r and f_b. A simple way is to let $f_r(\mathbf{a}(\mathbf{x}), t, \mathbf{u}) \approx W_r^u(\phi)\mathbf{a}(\mathbf{x})$ and $f_b(\mathbf{a}(\mathbf{x}), \mathbf{u}) \approx W_b^u \mathbf{a}(\mathbf{x})$, which result in the model presented in the main text.

4 DECOMPOSITION OF WEIGHT MATRICES UNDER THE IDEAL CONDITIONS

The ideal conditions require that the model should be stable given different $\mathbf{u}$, ϕ, and b, and the neurons should be complimentary for all $\mathbf{x}$ so as to encode the entire space in scope. More specifically and formally, there are three conditions to be met:

Condition 1: $\forall_{\mathbf{u}}\forall_{\phi}\|f_r(\mathbf{a}(\mathbf{x}),\phi,\mathbf{u})\| = c$ where c is a constant. It means that the drive for the on-plane displacement should not be selectively for plane orientation, $\mathbf{u}$, and motion orientation on the plane, ϕ.

Condition 2: In 3D navigation, $\forall_{\mathbf{u}}\|f_b(\mathbf{a}(\mathbf{x}),\mathbf{u})\| = c$. It also states the non-selectivity of plane orientation in the ideal case.

Condition 3: $\forall_{\mathbf{x}}\|\mathbf{a}(\mathbf{x})\| = c$. This is a strong constraint that reinforces the capability to represent the whole space. When the activity of an element in $\mathbf{a}$ (representing either a population of neurons or a single neuron) decreases, the activities of some other elements must increase.

If $W_r^u(\phi)$ and W_b^u are skew-symmetric, $(I + W_r^u(\phi)\delta r + W_b^u\delta b)^T(I + W_r^u(\phi)\delta r + W_b^u\delta b) \approx I$, i.e. $(I + W_r^u(\phi)\delta r + W_b^u\delta b)$ is a rotation matrix. As a result, $\exp(W_r^u(\phi)r + W_b^u b)$ is also a rotation matrix. Thus, **Condition 3** holds. Furthermore, the rotational mapping plays an important role in periodic activities during navigation.

Similar to (Gao et al., 2021), we find such skew-symmetric matrices by letting $\mathbf{a}(\mathbf{x}) = U\mathbf{e}(\mathbf{x})$ where U is a unitary matrix and $\mathbf{e}_k = \exp(iB_k\mathbf{x})$, so that $\|\mathbf{a}(\mathbf{x})\|^2 = (U\mathbf{e})^*(U\mathbf{e}) = \mathbf{e}^*\mathbf{e}$ is a constant. Note that B_k is a row vector. Then,

$$
\begin{aligned}
\mathbf{a}(\mathbf{x}+\delta\mathbf{x}) &= U\mathbf{e}(\mathbf{x}+\delta\mathbf{x}) \\
&= U\text{diag}(\exp(iB\delta\mathbf{x}))e(\mathbf{x}) \\
&= U\text{diag}(\exp(iB\delta\mathbf{x}))U^*\mathbf{a}(\mathbf{x}) \\
&\approx U(I+\text{diag}(iB\delta\mathbf{x}))U^*\mathbf{a}(\mathbf{x}) \\
&= (I+U\text{diag}(iB\delta\mathbf{x})U^*)\mathbf{a}(\mathbf{x}) \\
&= (I+U\text{diag}(iBM^u\boldsymbol{\gamma}_t)U^*\delta r + U\text{diag}(iB\mathbf{u})U^*\delta b)\mathbf{a}(\mathbf{x})
\end{aligned} \tag{S8}
$$

Consequently,

$$
\begin{aligned}
W_r^u(\phi) &= U\text{diag}(iBM^u\boldsymbol{\gamma}_t)U^* \\
W_b^u &= U\text{diag}(iB\mathbf{u})U^*
\end{aligned} \tag{S9}
$$

For **Condition 1** and **Condition 2** to hold, $\|W_r^u(\phi)\mathbf{a}(\mathbf{x})\|$ and $\|W_b^u\mathbf{a}(\mathbf{x})\|$ needs to be constant.

$$
\begin{aligned}
\|W_r^u(\phi)\mathbf{a}(\mathbf{x})\|^2 &= \|\text{diag}(iBM^u\boldsymbol{\gamma}_t)\mathbf{e}(\mathbf{x})\|^2 = \sum_k (B_kM^u\boldsymbol{\gamma}_t)^2 \\
\|W_b^u\mathbf{a}(\mathbf{x})\|^2 &= \|\text{diag}(iB\mathbf{u})\mathbf{e}(\mathbf{x})\|^2 = \sum_k (B_kM^u\mathbf{u})^2
\end{aligned} \tag{S10}
$$

where B_k are rows of B. Because both $M^u\boldsymbol{\gamma}_t$ and $\mathbf{u}$ are unit vectors (so that they have constant norms), **Condition 1** and **Condition 2** will hold if the rows of B form a tight frame of the space, i.e. $\sum_k(B_k\mathbf{v})^2 = A\|\mathbf{v}\|^2$ for a positive real number A and any $\mathbf{v}$. A set of solutions is the vertices of any platonic solid with its center of gravity at the origin. The simplest platonic solid is a tetrahedron, and a corresponding static

unitary matrix is easily found s.t. the resulting weight matrices are always skew-Hermitian:

$$B = b_0 R_0 \begin{pmatrix} 2\sqrt{2}/3 & 0 & -1/3 \\ -\sqrt{2}/3 & \sqrt{6}/3 & -1/3 \\ -\sqrt{2}/3 & -\sqrt{6}/3 & -1/3 \\ 0 & 0 & 1 \end{pmatrix}, \quad U = \frac{1}{2} \begin{pmatrix} 1 & 1 & 1 & 1 \\ 1 & \exp(-2i\pi/3) & \exp(i\pi/3) & -1 \\ 1 & \exp(i\pi/3) & \exp(-2i\pi/3) & -1 \\ 1 & -1 & -1 & 1 \end{pmatrix} \tag{S11}$$

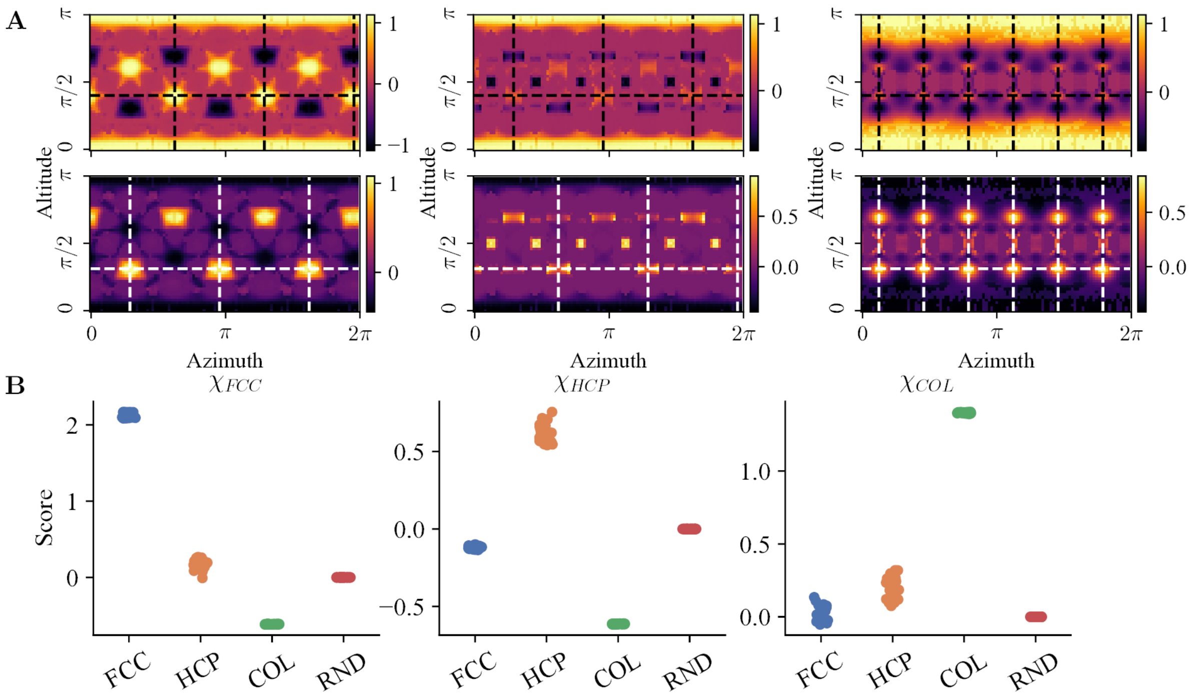

Figure S1. (A) The planar symmetry analysis of FCC (left), HCP (middle), and COL (right). Top, hexagonal grid score map; bottom, squared grid score map. The horizontal black dashed lines correspond to $72°$ and the horizontal white dashed lines correspond to $56°$. For HCP and FCC, two adjacent vertical lines are separated by $120°$, and the offset between vertical white lines and black lines is $60°$. Two nearby vertical lines in COL's maps are $60°$ apart. The intersections of horizontal and vertical dashed lines indicate local maxima. **(B)** The FCC, HCP, and COL structure scores for the 4 prototypes. $n = 30$ for each score.

REFERENCES

Finkelstein, A., Derdikman, D., Rubin, A., Foerster, J. N., Las, L., and Ulanovsky, N. (2015). Three-dimensional head-direction coding in the bat brain. *Nature* 517, 159–164. doi:10.1038/nature14031

Finkelstein, A., Las, L., and Ulanovsky, N. (2016). 3-D Maps and Compasses in the Brain. *Annual Review of Neuroscience* 39, 171–196. doi:10.1146/annurev-neuro-070815-013831

Gao, R., Xie, J., Wei, X.-X., Zhu, S.-C., and Wu, Y. N. (2021). On Path Integration of Grid Cells: Isotropic Metric, Conformal Embedding and Group Representation. *arXiv:2006.10259*
